# Day and night heat stress trigger different transcriptomic responses in green and ripening grapevine (*vitis vinifera*) fruit

**DOI:** 10.1186/1471-2229-14-108

**Published:** 2014-04-28

**Authors:** Markus Rienth, Laurent Torregrosa, Nathalie Luchaire, Ratthaphon Chatbanyong, David Lecourieux, Mary T Kelly, Charles Romieu

**Affiliations:** 1 Fondation Jean Poupelain, 30 Rue Gâte Chien, Javrezac 16100, France; 2 Montpellier SupAgro-INRA, UMR AGAP-DAAV & UMT Genovigne, 2 place Pierre Viala, Montpellier 34060, France; 3 INRA, UMR LEPSE, 2 place Pierre Viala, Montpellier 34060, France; 4 INRA, ISVV, UMR EGFV 1287, 210 chemin de Levsotee, Villenave d’Ornon F-33140, France; 5 Laboratoire d’Oenologie, UMR1083, Faculté de Pharmacie, Université Montpellier 1, Montpellier 34093, France; 6 INRA, UMR AGAP-DAAV, 2 place Pierre Viala, Montpellier, Cedex 02 34060, France

## Abstract

**Background:**

Global climate change will noticeably affect plant vegetative and reproductive development. The recent increase in temperatures has already impacted yields and composition of berries in many grapevine-growing regions. Physiological processes underlying temperature response and tolerance of the grapevine fruit have not been extensively investigated. To date, all studies investigating the molecular regulation of fleshly fruit response to abiotic stress were only conducted during the day, overlooking possible critical night-specific variations. The present study explores the night and day transcriptomic response of grapevine fruit to heat stress at several developmental stages. Short heat stresses (2 h) were applied at day and night to vines bearing clusters sequentially ordered according to the developmental stages along their vertical axes. The recently proposed microvine model (DRCF-Dwarf Rapid Cycling and Continuous Flowering) was grown in climatic chambers in order to circumvent common constraints and biases inevitable in field experiments with perennial macrovines. Post-véraison berry heterogeneity within clusters was avoided by constituting homogenous batches following organic acids and sugars measurements of individual berries. A whole genome transcriptomic approach was subsequently conducted using NimbleGen 090818 Vitis 12X (30 K) microarrays.

**Results:**

Present work reveals significant differences in heat stress responsive pathways according to day or night treatment, in particular regarding genes associated with acidity and phenylpropanoid metabolism. Precise distinction of ripening stages led to stage-specific detection of malic acid and anthocyanin-related transcripts modulated by heat stress. Important changes in cell wall modification related processes as well as indications for heat-induced delay of ripening and sugar accumulation were observed at véraison, an effect that was reversed at later stages.

**Conclusions:**

This first day - night study on heat stress adaption of the grapevine berry shows that the transcriptome of fleshy fruits is differentially affected by abiotic stress at night. The present results emphasize the necessity of including different developmental stages and especially several daytime points in transcriptomic studies.

## Background

Agricultural systems are vulnerable sectors to climatic variability and global warming. Drawing on the output from several simulation models, global mean surface temperature will rise between 1°C and 4.5°C, depending on future industrial emissions. The most optimistic estimates point to a 1.8 – 2.5°C warming by the middle of the next century [1,2]. Despite their multiple adaptive responses, most plants suffer reduced productivity when exposed to prolonged elevated temperatures [3,4]. The reasons for this decline are not fully understood on a molecular and physiological basis yet, but many studies in the current literature have been conducted to further elucidate this subject [3].

Increasing temperature is altering yields and quality of important annual global crops such as potatoes, rice, maize and wheat [5-7] in addition to perennials such as the grapevine, almonds, apples, oranges and avocados [8]. The most important changes in fruit production are predicted to occur only at the end of the 21^st^ century [9,10] leaving time for growers and breeders to adapt cultivation systems, change varieties or move to different climatic zones.

The grapevine is one of the most cultivated fruits with a total global surface area of 7.6 million hectares under vines, where most of it is processed to wine, leading to a global production of 265 million hectoliters [11]. Climate change, and in particular temperature increases have led to an alteration of wine quality and typicity in many growing regions over recent years [12-14]. This temperature increase will require varietal adaptations within traditional wine growing regions [15] but will nonetheless significantly reduce the suitable area for vine growing [16]. The principal modifications in the grapevine berry due to elevated temperatures occur during the ripening phase, resulting, for example in increased malic acid respiration leading to a drop in total acidity and increased pH [17-20]. Sugar concentration is usually promoted by high temperatures [21] leading as consequence to undesirably high alcohol levels. This combination of circumstances leads to poorly balanced wines that are microbiologically unstable with reduced aging potential and varietal aroma [22,23]. It has also been shown that berry size and weight at harvest are reduced by temperatures exceeding 30°C [24] in particular before the ripening phase [25,26]. Anthocyanin content in berries is usually lowered by high temperatures [27,28] due to impairment of biosynthesis [29] and/or accelerated degradation [29,30]. Frequently a shift in metabolites of the phenylpropanoid pathway is observed which seems to be highly temperature-sensitive. Tarara *et al*., 2008 [31] observed a change in anthocyanin composition with respect to malvidin-based derivates and Cohen *et al*., 2012 [32,33] reported a temperature-induced alteration in proanthocyanidin (PA) composition and concentration.

Several thermo-tolerance related genes have been recently characterized in grapevine [34-36]. Molecular and transcriptomic studies conducted on fruiting cuttings [35,37,38] led to the identification of genes directly involved in the heat stress response in the fruit. These studies provide new clues to the adaptation of the grapevine to high temperatures. However, the regulation of major metabolic pathways in response to heat stress within the fruit is by no means elucidated.

The grapevine berry undergoes marked physiological changes during its development [39,40]. Its growth pattern follows a double sigmoid curve [41] where the first phase is mainly dominated by cell division and enlargement [42], organic acid and tannin accumulation followed by a lag phase known as the herbaceous plateau. The transition between the lag phase and ripening is called véraison and is characterized by abrupt softening of the berry within 24 h. Most transcriptomic changes are triggered during this brief transition, before the resumption of berry growth [39]. The ripening phase can mainly be characterized by the accumulation of water and sugars, malic acid respiration and anthocyanin accumulation. The ripening growth period with its massive phloem unloading ceases simultaneously when hexose concentrations reach 1.1 M (ripe/maturity stage). Hexoses continue to concentrate by berry shriveling, due to evapotranspiration (over-ripening) [41,43,44].

Climatic chamber experiments are relatively complicated and costly with perennial plants like the grapevine, which has an annual reproductive cycle. Therefore, experiments are usually carried out in the field, where the fine control of temperature becomes obviously impossible, on the contrary to water availability. Biases introduced by fluctuations in the environment are difficult to circumvent and usually unquantified. Transient variations in direct or reflected light irradiance, air speed and moisture, may, through acting on stomatal conductance and plant surface temperature, erratically affect major physiological processes (respiration, photosynthesis) and thereby genes expression level. Additionally, unfavorable environmental conditions may amplify the noticeable asynchrony in berry ripening due to increasing berry competition for photoassimilates [45,46]. The statistical bias resulting from mixing unsynchronized berries probably masks many targeted effects in molecular studies. Here we use the L1 *gai1* (GA insensitive) mutant of Pinot Meunier L. [47,48] as a recently proposed model for grapevine research [49-52]. Its dwarf stature and continuous fructification along the main axis render it particularly suitable for experiments in climatic chambers.

Recent microarray screenings of cDNAs have shown that critical events in the program of berry development occur specifically at night [53]. Furthermore, the same study showed that day - night modulated transcripts differ to a large extent according to berry stage. For example, transcripts associated with secondary metabolism were mainly up-regulated at night in ripening berries, whereas cell wall synthesis and modification processes were enriched in night-induced genes at green stages. To the best of our knowledge long-term effects of moderate temperature gradients have retained most attention on fleshy fruits and their transcriptomic responses to abiotic stress have never been characterized during the night [38]. In those studies, plants had the time to adapt to their changed environments, which probably masked many heat-induced transient changes in gene expression critical for long term adaptation.

The present study is the first where whole plants grown in climatic chambers under precisely controlled cool conditions were subjected to a short but abrupt period of heat stress during day or at night. The microvine model enabled the application of this stress at several stages of berry development simultaneously. Changes in gene expression under heat stress were analyzed with whole genome 30 K microarrays (NimbleGen 090818 Vitis 12X) on green berries and two post-véraison stages. Two sets of gene annotations derived from Grimplet *et al*., 2012 [54] and from the NCBI RefSeq [55] database were used for functional annotations. Depending on the developmental stage, considerable differences in the response of berries to heat stress between the day and night were revealed, emphasizing the necessity to include night-time in further studies on abiotic stress in plants.

## Results and discussion

### Stress application and sampling protocol

A short stress period of two hours was applied to whole plants bearing berries at all reproductive stages from flowering to maturity, following an acclimatization period of ten days at constant day and night temperatures (22/12°C). The target heat stress temperature was set at 37°C for both day and night treatments which may appear as a rather moderate stress for grapevine, that would just impair photosynthesis by 17% [56]. Berries exposed to solar irradiance can reach temperatures 10°C above those of ambient air [57,58] and grapevine vacuolar proton-pumps, that play a predominant role in the energization of the tonoplast are thermostable up to 65°C [59,60]. However this temperature triggered maximal expression of the two heat shock proteins *At-HSP17.6* and *At-HSP18.*2 in *Arabidiopsis thaliana*[61,62] and several studies indicate that after two hours, a transcriptomic shift in the heat stress response occurs in other plant species [61,63].

**Figure 1:**
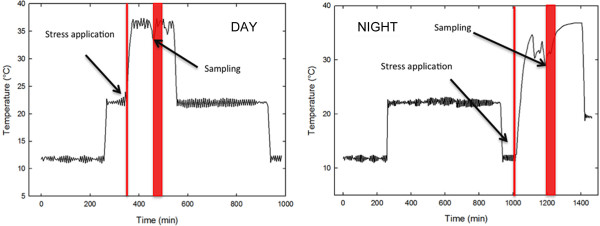
Temperature recordings in climate chambers during stress application and sampling for day and night treatment.

As Figure 1 illustrates, during the day, this temperature could be achieved within 15 min and remained fairly constant with a slight drop during sampling due to the opening of the chamber. During the night the rise in temperature took slightly more time owing to the lack of supplemental warming by the lighting system, which was switched off. The first stage of fruit development analyzed was composed of green berries (G) sampled during the first growth phase where malic acid accumulates at maximal rate as major osmoticum, while tartaric acid synthesis has already ceased (as illustrated by the small fruit size, the lack of hexoses and a ca. 50% load in malic acid: Table 1). The two consecutive developmental stages were composed of berries sampled in clusters at and after the onset of ripening, as estimated by pericarp softening. Due to a lack of synchronism in the ripening process, single berries were individually frozen and powdered in liquid nitrogen before sugar and organic acid HPLC analysis, in order to constitute two homogenous batches for RNA extraction, named VéraisonSugar (VS, 0.16 mol.Kg FW^-1^ hexoses) and VéraisonColor (VC, 0.36 mol.Kg FW^-1^ hexoses; Table 1), because no coloration could be detected in the VS samples. These respective values represented 1/7 and 1/3 of hexose concentration in ripe berries (not shown), indicating that sugar storage had just began in the VS samples, and proceeded at maximal rate in the VC ones. Malate breakdown was negligible between VS and VC, owing to the relatively cool temperature of the acclimation period. The 2 h 37°C period was obviously too short to detect the induction of malate breakdown by heat stress.

**Table 1:** Biochemical characteristics of extracted samples

**Stage**	**Treatment**	**Avg berry weight (g)**	**Hexoses (mol.kg.FW**^**-1**^**)**	**Malate (μEq.berry**^**-1**^**)**	**Tartrate (μEq.berry**^**-1**^**)**	**Total anthocyanins (μg.berry**^**-1**^**)**
Green Stage (G)	CD	0.54 ± 0.08	nd	150 ± 15	97 ± 8	nd
	TD	0.51 ± 0.04	nd	155 ± 10	101 ± 7	nd
	CN	0.39 ± 0.10	nd	145 ± 12	107 ± 7	nd
	TN	0.47 ± 0.03	nd	143 ± 17	102 ± 5	nd
Véraison Sugar (VS)	CD	1.7 ± 0.2	0.16 ± 0.06	290 ± 30	110 ± 10	nd
	TD	1.6 ± 0.3	0.21 ± 0.03	280 ± 22	108 ± 6	nd
	CN	1.3 ± 0.2	0.18 ± 0.03	275 ± 20	105 ± 8	nd
	TN	1.4 ± 0.2	0.12 ± 0.01	278 ± 17	103 ± 11	nd
Véraison Color (VC)	CD	1.6 ± 0.4	0.35 ± 0.02	262 ± 43	105 ± 8	4.2 ± 1.7
	TD	1.9 ± 0.4	0.36 ± 0.03	255 ± 39	103 ± 4	4.6 ± 3.3
	CN	1.6 ± 0.3	0.34 ± 0.03	265 ± 40	102 ± 6	8.4 ± 2.3*
	TN	1.5 ± 0.3	0.38 ± 0.02	258 ± 37	104 ± 6	2.9 ± 1.6*

CD: Control Day: TD: Treatment Day; CN: Control Night; TN: Treatment Night.

± standard deviation (n = 3).

*significant differences between treatment (p < 0.05).

Biochemical analysis confirmed that berries within the VC stage just started to synthesize anthocyanins (Table 1), with a noticeable delay following the onset of sugar storage. It is known that conditions prevailing during the night play an important role in grape berry composition particularly during ripening [64,65]. Heat treatment seemed to have reduced total anthocyanin content at night by factor 2.5 but not during day. This result, which is in accordance with long-term temperatures studies, conducted during the day [28,56], appears rather surprising given the short duration of stress application. A previous gene expression study showed activation of transcripts involved in secondary metabolism during the night in ripening berries, but not specifically for anthocyanin-related transcripts [53]. Since total anthocyanin content was generally very low in analyzed samples, the observed difference between stressed and control samples could be an analytical artefact.

### Main transcriptional variations induced by high temperature

**Figure 2:**
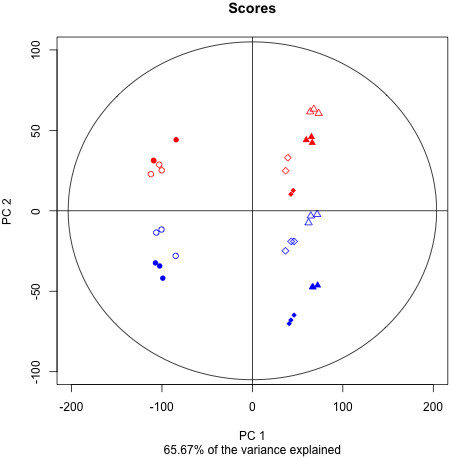
**Principal component analysis on normalized expression data.** Red: heat stress; Blue: control; Filled symbols: night; Empty symbols: day; Circles: Green Stage (G); Squares: VéraisonSugar (VS); Triangles: VéraisonColor (VC).

Principal component analysis of normalized gene expression data is presented in Figure 2. Despite the fact that berries were still green in the véraison sugars (VS) samples and their hexose concentration was only 1/7 of that expected at the ripe stage (not shown) pre- and post-véraison berries are clearly distinguished on PC1 accounting for 52% of the variation, which can be explained by the shift in the berry transcriptome during softening, before or at the very beginning of sugar accumulation [39,40]. PC2 explained 14% of the variation and accounts for changes in gene expression triggered by temperature. The variations due to temperature on PC2 were almost the same for all developmental stages.

A clear day - night separation could be detected with PC4 (Additional file 1) in the sense that the difference in gene expression between day and night were noticeably impaired by heat stress at all developmental stages. The expression data was consistent and reproducible between replicates and therefore considered reliable for further analysis.

**Figure 3:**
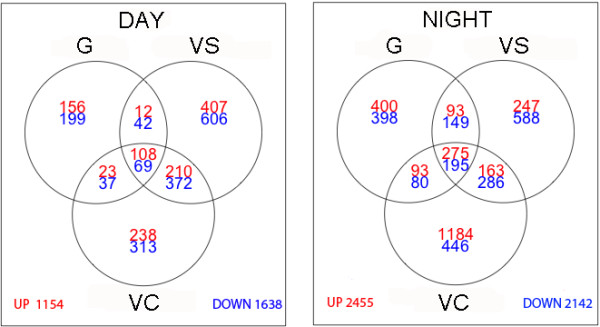
Venn Diagrams of up-or downregulated transcripts (fold change > 2; padj < 0.05) between control and heat stress at the different developmental stages separately (G: Green, VS: VéraisonSugar, VC: VéraisonColor) for DAY (left) and NIGHT (right).

All transcripts differentially expressed between control and heat stress in at least one of the three developmental stages and time points were extracted (fold change >2, pval adj <0.05), yielding a total of 5653 heat modulated genes (Additional file 2). Venn diagrams (Figure 3) show the number of transcripts modulated by stress at all stages separated by day and night. Greater changes in gene expression were triggered at night, as illustrated by a 1.6 fold increase in the total number of genes induced or repressed at night. It can be argued that the absolute applied temperature in the heat treatments was theoretically the same for both day and night and thus the temperature gradient between control and heat-stressed plants was greater at night thereby inducing larger modifications. However, this seems quite unlikely since, due to the previously described technical difficulties, stress at the target temperature was in fact shorter at night, and this increase in stress-modulated genes at night did not hold for VS. Interestingly a dramatic five-fold increase in genes triggered by temperature at night was observed at VC. Analyses of functional categories of heat-modulated genes are illustrated in Additional files 3 and 4. Temperature stress response, heat shock protein (HSP)- mediated protein folding and HSP 70 related categories were induced under in all stages at day and at night which illustrates that their temperature regulation prevails over developmental or circadian regulation. Interestingly these categories were least responsive at night in VS. On the other hand, stage or/and time point specific heat induction can be observed for some categories, such as cell wall modification and metabolism in G or xyloglucan modification only at night in G and VC (Additional file 3).

Amongst heat-repressed transcripts a night specific repression at VS of stilbenoid and phenylalanine metabolism and synthesis can be remarked and confirms observations made in a a previous study [53] where a night up-regulation of these pathways was observed under controlled conditions. On the other hand pathways such as terpenoid biosynthesis and metabolism were downregulated only at day in G (Additional file 4).

### Identification of similarly regulated transcripts in all conditions

**Figure 4:**
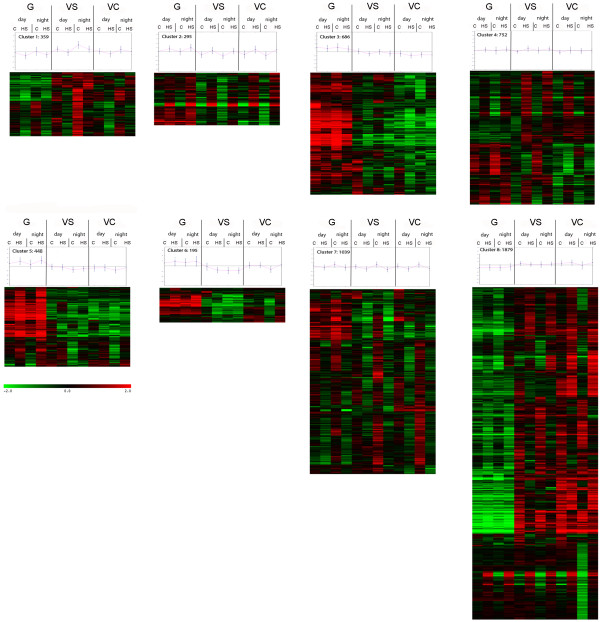
**Cluster with their centroid graphs identified by hierarchical clustering on averaged and mean centered expression values of all modulated transcripts.** Stages are ordered according to developmental stage form the left: G (Green), VS (VéraisonSugar), VC (VéraisonColor), day and night and Control (C) and Heat Stress (HS).

In order to identify patterns of gene expression commonly regulated during both daytime and night-time heat stress, the 5653 detected transcripts were allocated into 8 clusters by hierarchical clustering (Figure 4) before analyzing the relative enrichment of functional categories (Additional file 5).

Transcripts consistently induced by heat stress during all developmental stages were mainly allocated to cluster 2 and 4. In cluster 4, the heat stress response occurred mainly at VS and was more subtle than in cluster 2. The HSP (Heat Shock Protein) – mediated protein folding and temperature stress functional categories were enriched (Cluster 4; Additional file 5), indicating that the main heat stress associated transcripts are triggered by temperature independently of developmental stage and day time. Conversely, other functional categories exhibited clear heat stress regulation only at specific stages. For example cell wall modification related processes prevailed in cluster 5, which includes transcripts mainly modulated by heat stress in green berries and subsequently repressed in later stages. Transcripts consistently repressed by temperature can be found in cluster 1 and clusters 7. In the latter, down-regulation appears to be less pronounced and enriched categories of allocated transcripts were principally related to hormone signaling, primary metabolism and some secondary metabolism. This suggests that genes within these families respond less or at a slower rate to temperature increases.

Some clusters can be attributed to genes that showed clear heat stress regulation only at specific stages. For example cluster 5, which comprises transcripts mainly modulated by heat stress in green berries and subsequently repressed during ripening is dominated by modification of cell wall-related processes. Clusters 3, 6 and 8 are dominated by developmentally-regulated genes. Cluster 6 comprises genes modulated between G and VS that become more responsive to diurnal changes and stress at VC. Transcripts within this cluster can be attributed to the biosynthetic pathway of flavonoids and xyloglucan modification, which were both considerably over-represented. This first global analysis demonstrates that functional categories related to processes other than a response to heat stress response do exhibit a very different thermal modulation according to developmental stage.

### High temperature induced heat shock related genes whose modulation varied little along stages and day time

**Figure 5:**
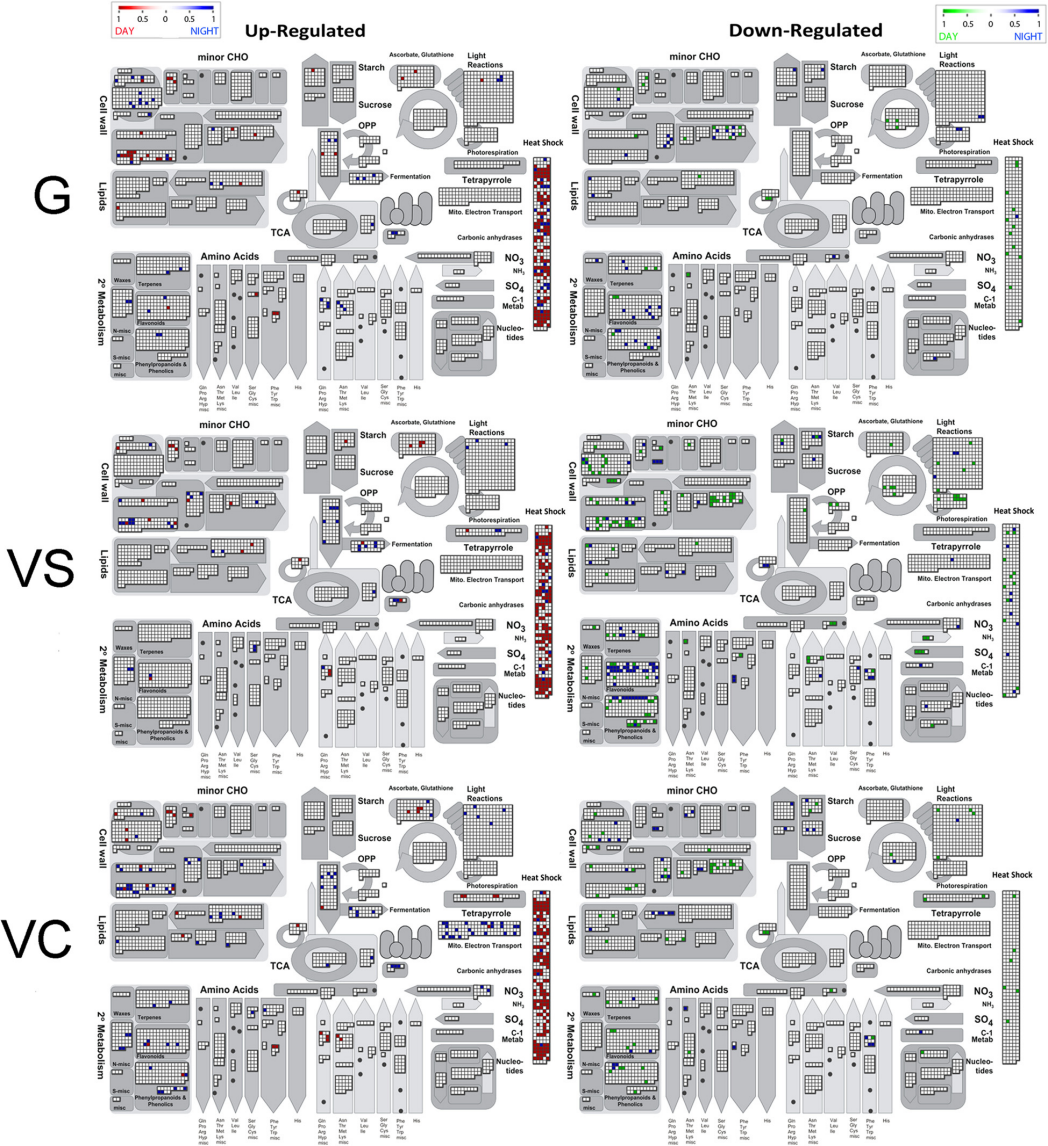
**MapMan overview of day and night modulated transcripts at the three different stages: G (Green), VS (VéraisonSugar) and (VC) (VéraisonColor).** Left: up-regulated transcripts; Red: day and night modulated; Blue: night-specific; Right: down-regulated transcripts; Green: day and night modulated, Blue: night-specific. Scale in log_2_ control/stress.

Functional categories within heat stress-induced transcripts (Additional file 3) were mainly related to abiotic/temperature stress response and Heat Shock Protein (HSP)/Chaperone - mediated protein folding. This was consistent at all stages during the day and at night. A more detailed illustration of heat-modulated transcripts is given in the MapMan graph (Figure 5). This figure differentiates night-specific genes from those modulated by heat stress in an experiment, which only considers daytime stress. In regards to heat shock protein category it can be observed that most of the heat shock responsive genes were heat induced during the day and at night, whereas only a small number of the latter were specifically night-modulated which was most apparent in green berries (G).

Similar categories were shown to be modified by temperature in a previous study with fruiting cuttings [38]. The short heat stress period of 2 h in the present study presumably enhanced the induction of these transcripts in which over-expression was not observed with longer temperature treatments in other studies where plants started to adapt to their changed conditions [66-68]. Heat-shock proteins (HSPs)/chaperones are responsible for protein folding, assembly, translocation and degradation in many normal cellular processes; they stabilize proteins and membranes, and can assist in protein refolding under stress conditions thus preventing the formation of abnormally folded protein structures [69]. HSPs have been shown to be a prerequisite in plant thermo-tolerance [34,70-72] and other abiotic stresses [73].

The expression of HSPs in response to various stimuli is regulated by heat shock transcription factors (HSFs) [72]. In this study several HSFs were induced upon heat stress, most of them regularly amongst all conditions and stages. Yet, some displayed quite interesting modulation patterns. A HSF (*VIT_04s0008g01110*; cluster 8) was consistently up-regulated by heat stress, but this induction was more pronounced at night than during the day, irrespective of developmental stage. The latter locus is annotated *HSFA6B* according to Grimplet *et al*., 2012 [54] and *HSF30*-like according to RefSeq [55], and was previously identified and named *VvHsfA*2 in heat stressed Cabernet Sauvignon berries [35]. A heat shock transcription factor *B2B* (*VIT_02s0025g04170*; cluster 4) involved in pathogen resistance in *Arabidopsis thaliana*[74] was also induced at all stages by thermal stress during the night only. Several transcripts coding for members of the family of ethylene responsive transcription factors (ERFs), which are thought to intervene in the regulation of abiotic stress response, acting upstream of HSFs [75,76] exhibited a very distinct modulation: *VIT_04s0008g06000*, *VIT_18s0001g03120; VIT_18s0001g05850; VIT_16s0013g00980* and *VIT_16s0013g01000* were all activated by heat stress but only at night in green berries. This stage-specific temperature response of ERFs is amplified by an over-representation of this functional category in cluster 5 (Figure 4; Additional file 5).

**Figure 6:**
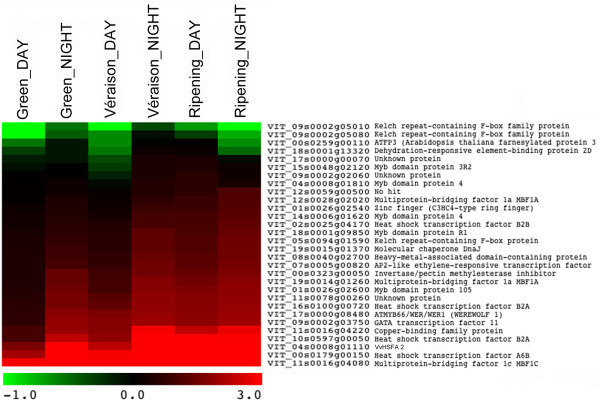
**Expression matrix of MBF1c regulon transcripts from *Arabidopsis thaliana *identified in NimbleGen 090818 Vitis 12X microarrays.** Scale is in log_2_ change between control and heat treatment.

Amongst heat shock transcription factors, we also detected *MBF1c* (*VIT_11s0016g04080*; cluster 8) induced at all stages and *MBF1a* (*VIT_19s0014g01260*; cluster 8) at night in VC. *MBF1c* did not show differences in day/night stress regulation in VS and VC, but in green berries, its response was more than two fold greater at night than during the day. *MBF1c* acts upstream to salicylic acid, ethylene and trehalose in the heat stress response of *Arabidopsis thaliana*[77,78] where its regulon was previously characterized [77]. The putative *Vitis vinifera* orthologs of the genes inside the *Arabidopsis MBF1c* regulon were identified amongst those probed by the NimbleGen 090818 Vitis 12X microarrays. The expression matrix illustrated in Figure 6 confirms that most of these transcripts were actually induced by heat stress in grapevine berries as well. However their response was less significant in the green berry, with even inversions in some cases.

The present results suggest that the expression of some heat shock transcription factors is correlated with the temperature gradient, which was greater for the night heat stress treatment. Conversely, the regulation of other heat shock transcription factors seems to be triggered as soon as heat stress is experienced by the plant, regardless of the temperature gradient, berry stage or time of day.

Galactinol (GOL) and other raffinose (RFO) oligosaccharides accumulate in response to heat stress in plants and can act as osmoprotectants in cells [79]. Galactinol synthase (*GOLS*) catalyses the first committed step in the RFO biosynthetic pathway, synthesizing galactinol from UDP-_D_-galactose and *myo*-insositol. It has been identified and characterized previously as day heat-responsive gene in Cabernet Sauvignon L. berries exposed to elevated temperatures [35]. Here, seven transcripts annotated as *GOLS*[54] or glycogenin-2 [54] were detected. Only two of these showed consistent induction in response to heat stress at several stages (*VIT_07s0005g01970*; cluster 2 and *VIT_14s0066g02350*; cluster 5) whereas *VIT_07s0005g01970* corresponds to the *VvGOLS1* gene characterized in previous temperature studies by Pillet *et al*., 2012 [35].

The same inconsistent regulation of transcripts annotated as raffinose synthase [54] or as galactinol-sucrose galactosyltransferase in the RefSeq [55] annotation was observed: *VIT_17s0000g08960* was allocated to cluster 2 thus induced by stress at several stages whereas *VIT_14s0066g00810* was assigned to cluster 1 exhibiting tendencies of down-regulation. Carbonell-Bejerano *et al.,* 2013 [38] observed the induction of an osmotin transcript (*VIT_02s0025g04340*) indicating its putative function in activating osmoprotection in response to elevated temperatures. Conversely, in this study, this transcript and three other osmotin-coding genes were down-regulated by heat stress at VS during the day, which brings into question the actual role of this gene in response heat stress in grapevine fruits.

### Phenylpropanoid and in particular anthocyanin-related transcripts are impacted by short heat stress

Phenolic compounds are major wine quality determining substances derived via the phenylpropanoid pathway. They are largely responsible for the color and astringency of wines and are attributed to various physiological benefits associated with moderate wine consumption [80]. Phenolic compounds comprise a range of structural classes such as lignins, phenolic acids, flavonoids and stilbenes [81]. The MapMan overview in Figure 5 illustrates the importance of daytime and developmental stage on the heat response of transcripts within secondary metabolism where phenylpropanoid and flavonoid pathways are mainly temperature-affected at VS at night. Several phenylalanine ammonia-lyase (*PAL*) coding transcripts (*VIT_16s0039g01100, VIT_16s0039g01120, VIT_16s0039g01130, VIT_16s0039g01240*; cluster 7), the key enzyme of the phenolpropanoid pathway [82] were repressed by high temperature during the night at VS only. The same pattern could be detected for chalcone synthase (*CHS*), the first committed enzyme in flavonoid biosynthesis [83]; three *CHS*s transcripts were strongly down-regulated by heat stress at VS at night (*VIT_14s0068g00930, VIT_14s0068g00920, VIT_16s0100g00860*). *VIT_16s0100g00860* is probably not correctly annotated in Grimplet *et al*., 2012 [54] since it is named stilbene synthase (*STS*) in RefSeq [55]. This annotation problem is probably due to the high number of *STS* in the grapevine reference genome (PN4002), its evolution and hence high similarity to *CHS. STS*s and *CHS*s are both members of the type III polyketide synthases family, whereas *STS*s play an important role in the adaptation of plants to abiotic stresses [84].

Transcripts involved in flavonoid synthesis were found to be repressed by heat stress at VS, such as UDP-glucose:flavonoid 7-O-glucosyltransferase transcripts (*VIT_05s0062g00660, VIT_05s0062g00700, VIT_05s0062g00270, VIT_05s0062g00710, VIT_05s0062g00350*), several STS coding transcripts such as *VvSTS18* (*VIT_16s0098g00860*) [85], which is not correctly annotated in Grimplet et al., 2012 [54] and RefSeq [55], and a resveratrol synthase (VIT_16s0100g01070).

Proanthocyanidins (PAs) are polymers of flavan-3-ol subunits often called condensed tannins that also derive from the phenylpropanoid pathway. They protect plants against herbivores, and UV radiation; they are important quality components of many fruits and constitute the majority of wine phenolics [86]. Two enzymes, leucoanthocyanidin reductase (*LAR*) and anthocyanidin reductase [87] can produce the flavan-3-ol monomers required for the formation of PA polymers [88]. Indications exist that increased temperature enhances the production of PA in grape berries [32,33]. The present study could not confirm these results since a *LAR* transcript (*VIT_01s0011g02960*) was found to be repressed by heat stress at VS at night in addition to an *ANR* (*VIT_00s0361g00040*) in green berries at night.

Anthocyanins belong to the group of flavonoids which are plant pigments responsible for red, blue and purple color of plant tissue and whose accumulation is often induced by abiotic stress [89,90]. Several studies report an increase in their accumulation during berry ripening in low temperature conditions and *vice versa*[25,31,91]. However, not all genes involved in anthocyanin biosynthesis showed unambiguous repression by high temperature in previous field studies [29,30], contrary to detached fruits *in vitro*, [92]. In field experiments, Yamane *et al*., 2006 [93] found *VvMYBA1*, which controls anthocyanin biosynthesis in grapes [94-97] to be repressed by heat, however, this was not confirmed in fruiting cuttings despite repression by temperature of several anthocyanin transporters (*VvanthoMATE/VvAM1* and *VvAM3*) downstream to *VvMYBA1*[38].

Several transcripts of the late anthocyanin biosynthesis pathway were actually repressed by temperature in this study. Heat repression at night was more evident as was their nighttime expression compared to daytime expression. *VvMYBA1* isogenes (*VIT_02s0033g00380, VIT_02s0033g00410, VIT_02s0033g00440*; cluster 8) were down-regulated by heat stress only at VS, both during the day and at night, consistently with Glutathione S-transferase *GST* (*VIT_04s0079g00690*), Caffeoyl-CoA O-methyltransferase (AOMT1; *VIT_01s0010g03510*) and *VvanthoMATE*3, which *s*pecifically mediates the transport of acylated anthocyanins*.* All these transcripts were shown to be correlated with anthocyanin accumulation and trans-activated upon ectopic expression of *VvMYBA1*[89-91]. Surprisingly, *UFGT* (UDPglucose: flavonol 3-O-glucosyltransferase) at the last step of anthocyanin biosynthesis [94,98,99] did not correlate with the expression of *VvMYBA*1, and even appeared to be heat-induced during the day. The expression profiles of these genes were duly validated by real time PCR in order to confirm microarray data (Additional file 6).

This immediate response by processes involved in secondary metabolism is remarkable given the brevity of the heat stress applied and has not been observed hitherto in temperature experiments on grapevine berries. It demonstrates that changes in gene expression involving secondary metabolism occur at the onset of sugar loading, before any changes in coloration can be detected. It has been shown here that coloration may be significantly delayed as compared to hexose accumulation, and that transcriptomic effects are usually masked by berry heterogeneity within bunches around véraison. Presumably the use of reconstructed groups of samples after single berry biochemical analyses and the inclusion of night sampling enabled the observation above to be made. Further work is required to validate that *UFGT* may escape from the *VvMYBA1* regulon upon heat stress at the very early stages of berry ripening which may however be consistent with its role as quercetin-glucosyl-transferase [96].

### Evidence of a reduction in aromatic potential in grapevine berries exposed to high temperatures

Low temperatures favor aroma production in grapevine berries especially during ripening [64,65]. This is well manifested in the enhanced aromatic potential of cool climate white wines [12] made from cultivars such as Gewürztraminer, Sauvignon Blanc or Riesling where major aroma compounds are isoprenoids, notably monoterpenes. Consequently, elevated temperatures potentially reduce the aromatic potential of grapevine fruit [100,101]. The present study supports this observation from a transcriptional point of view. High temperatures impaired the expression of 1-deoxy-D-xylulose-5-phosphate synthase transcripts (*VIT_11s0052g01730, VIT_11s0052g01780*; cluster 7) required for isopentenylpyrophosphate (IPP) synthesis, the universal precursor for the biosynthesis of terpenes [102]. Several transcripts coding for geraniol 10-hydroxylase, an enzyme thought to play an important role in indole alkaloid biosynthesis [103], were down-regulated at night by high temperatures at VS. However these transcripts are annotated as cytochrome P450 in the RefSeq [54] database. Further evidence for the impairment of terpene production arises from the repression at all growth stages of transcripts coding (-)-germacrene D synthase, a sesquiterpene synthase characterized recently in grapevine berries [104] (*VIT_19s0014g02560, VIT_19s0014g02590*; cluster 8, and *VIT_19s0014g04840, VIT_19s0014g04880*; cluster 3), in addition to linalool synthase (VIT_00s0271g00060; cluster 3; annotated nerolidol synthase in RefSeq [55]) which catalyses the formation of the acyclic monoterpene linalool from geranyl pyrophosphate [105].

Carotenoids play also an important role in wine flavor since they can be cleaved and their concentration is directly linked to C_13_-norisprenoids [106]. The C_13_-norisoprenoids identified in wine with important sensory properties are TCH (2,2,6-trimethylcyclohexanone), β-damascenone, β-ionone, vitispirane, actinidiol, TDN (1,1,6-trimethyl-1,2-dihydronaphthalene), Riesling acetal and TPB (4-(2,3,6-trimethylphenyl)buta-1,3-diene) [107]. The first committed step in carotenoid biosynthesis is the production of 40-carbon phytoene from the condensation of two geranylgeranyl pyrophosphate (GGPP) molecules, catalyzed by the phytoene synthase (*PSY*) enzyme. As a result of thermal stress, a repression of a *GGP synthetase 1* (*VIT_18s0001g12000*; cluster 7) was observed at night in G and VS.

### Indication that heat stress delays fruit ripening

Increasing evidence suggests that the hormone abscissic acid (ABA) is involved in the initiation of berry ripening and sugar accumulation [108,109]. The plastidial enzyme 9-cis-epoxy-carotenoid dioxygenase (*NCED*) catalyses the first committed step in ABA biosynthesis by producing xanthoxin [110]. Several *NCED* isogenes were consistently repressed by heat stress at VS and VC, in addition to two putative ABA receptors (VIT_08s0058g00470; cluster 1 and VIT_15s0046g01050; cluster 7). High temperatures have been reported to delay and even stop ripening and sugar accumulation in several experiments [26,28,111-113]. The present indications of a decrease in ABA synthesis and thus a delay in the onset of ripening are supported by the repression of different sugar transporters (*STP*s; *VIT_09s0018g02060, VIT_13s0019g01320, VIT_13s0019g01400* cluster 8 and *VIT_00s0181g00010*, cluster 1). They were only repressed in the first véraison stage (VS) and even appeared to be induced on the later stage (VC), especially at night. These results thus confirm a putative delay of ripening induced by high temperatures, but only when heat is applied at the very early stages of sugar accumulation. At the stages when sugars accumulate at maximal rate, the molecular data suggests that sugar accumulation is accelerated by elevated temperatures. This is in agreement with general observations by viticulturists that moderately warm temperatures favor sugar accumulation during ripening [21,112,114] leading to wines higher in alcohol in warm climates and seasons. In a previous study on fruiting cuttings ABA levels were significantly increased by high temperature after 45 but not after 14 days of post-véraison heat treatment [38]. This is consistent with our results where the repression of ABA synthesis genes in berries at véraison is inversed at the more developed stages. This is the first time where molecular data in the same experiment suggests a delay in and an acceleration of sugar accumulation in berries in response to high temperatures depending on berry stage where stress is applied. This illustrates the importance of precise stage selection of post-véraison berries if a precise deciphering of molecular changes is to be obtained.

### Proline biosynthesis seems to be activated upon heat stress whereas other genes involved in amino acids biosynthesis did not show consistent modulation

Proline (Pro) and Arginine (Arg) constitute up to 70% of total nitrogen in grapevine berries at maturity. Pro accumulation is induced during ripening [115,116] and is amplified in berries exposed to higher temperatures [19]. Pro accumulation has been associated with various stresses in eubacteria, protozoa, marine invertebrates and plants. In this study a transcript coding for a delta 1-pyrroline-5-carboxylate synthetase (*P5CS*; VIT_15s0024g00720; cluster 4) was up-regulated by heat stress during the day at VS. *P5CS* is a bifunctional enzyme catalyzing the activation of glutamate by phosphorylation and the subsequent reduction of the labile intermediate c-glutamyl phosphate [117,118]. This is consistent with the fact that a glutamate synthase (*VIT_12s0055g00620*; cluster 5) and several glutamate receptors isogenes were up-regulated by heat treatment at G at night. These observations concur with the role of Pro in the adaptation of the berry to a wide range of abiotic stresses (including water deficit); these transcripts were induced by water deficit in Cabernet Sauvignon and Chardonnay berries in a previous study [119].

Most of other amino acid-related transcripts were repressed by high temperatures. This is in agreement with current understanding where a modification of amino acid content by heat stress is generally not observed. Carbonell-Bejerano *et al*. [38] reported no common pattern of amino acid accumulation due to the effect of high temperature. They did find that the concentration of some amino acids increased by high temperatures namely, tyrosine, valine, methionine and ornithine in fruiting cutting at 14 days after the start of treatment, but that this difference disappeared at 45 days. In this study, transcripts associated with amino acid synthesis observed in microvine berries did not confirm the above findings, as most of genes related with the biosynthesis of the above-mentioned amino acids were either not detected or were found to be repressed by heat stress. However, an alanine-glyoxylate aminotransferase (*VIT_08s0058g00930*; cluster 7) and three aspartate aminotransferases (*VIT_08s0058g01000*; cluster 5, *VIT_04s0008g04250*; cluster 4 and *VIT_12s0055g00920*; cluster 8) transcripts were observed to be highly up-regulated by heat stress at all stages, with the exception of G during the day.

The tripeptide glutathione comprising the amino acids Gly, Cys and Glu is often associated with oxidative stress, acting as a reactive oxygen species (ROS) scavenger in plants [120]. Many glutathione S-transferases coding transcripts were modulated by heat stress but a clear pattern did not emerge. In ripening berries stress-induction was evident only at night, which calls into question its role in the heat shock response of the berry. In temperature experiments on fruiting cuttings down-regulation of two transcripts coding for cationic amino acid transporters, *VIT_10s0003g04540* and *VIT_13s0073g00050*, thought to be involved in cellular import of amino acids [121], is reported [38]. The authors hypothesized that this repression could compensate for probable greater transport activity resulting from higher membrane fluidity at elevated temperatures. The present study would partially confirm this as it was observed that the same isogenes were allocated to cluster 7, and thus down-regulated at G and VS stages only at night. Additionally, and pointing in the same direction, several histidine/lysine transporter transcripts were repressed by stress mainly at night and at all developmental stages.

### Malic enzyme and mitochondrial transporters were activated by high temperatures

Temperature stimulates the respiration of malic acid in grapevine fruit leading to a decrease in total acidity under warm climatic conditions [17-20]. However, the trigger and principal pathways of malic acid degradation have not been entirely resolved [122-125]. Degradation can take place by oxidation to pyruvate via malic enzyme (ME), with pyruvate entering the TCA cycle either directly [126], or following ethanol recycling via the pyruvate dehydrogenase bypass [39]. Alternatively, oxaloacetate (OAA) formed by malate dehydrogenase (MDH) constitutes the entry point for neoglucogenesis, before PEP formation catalyzed by phosphoenolpyruvate carboxykinase (PEPCK) [123]. PEPCK enzyme activity [127] and transcript abundance [39,128] are increased in post-véraison grapes. However, neoglucogenesis is an energy-consuming process, which would be inhibited by stress. Under heat stress, a *PEPCK* isogene (*VIT_00s2840g00010*; cluster 7) was down-regulated at all stages. Furthermore, simultaneous thermal up-regulation of *MDH* and *ME* coding transcripts (*VIT_19s0014g01640* and *VIT_00s0279g00080*, cluster 4) was observed during the day and at night at VS, when malate breakdown occurs at maximal rate. Two additional *ME*s isogenes (*VIT_04s0008g00180, VIT_02s0012g02460*; cluster 8) were increasingly up-regulated at all stages towards ripening, but only at night. This indicates that at elevated temperatures, the oxidation of MA by ME and MDH is favored when compared to the neoglucogenesis pathway. Moreover, two alcohol dehydrogenase transcripts (*VIT_04s0044g01120, VIT_04s0044g01130*; cluster 8, annotated as *ADH2*[54] or *ADH1*[55], the first ripening-related enzyme found in grape [123] were activated by heat stress at night at all stages, while an *ADH* (VIT *04s*_0023g02810, cluster 8) was induced at night in VS and VC. Ethanolic fermentation of malic acid scavenges two protons from the cytosol, thereby allowing the efflux of vacuolar malic acid to transiently exceed the capacity of its respiration and neoglucogenesis, during warm nights. Aerobic fermentation enhanced by elevated temperatures in ripe detached berries [129] may represent a vital adaptation to heat stress once malate breakdown has been induced in parallel with increased permeability of the tonoplast [130].

The supposed function of tonoplast dicarboxylate transporters (TDT) in malic acid degradation, transporting MA from the vacuole to the cytoplasm where it can be catabolized, was recently proposed [123,128]. Aluminium-activated malate transporters (ALMT) are involved in vacuolar malate transport in *Arabidopsis thaliana*[131] and a truncated isogene has been associated with low fruit acidity in apples [132]. ALMT transcripts showed consistent up-regulation in ripening berries [53].

*VvALMT9* acts as a typical inward rectifying channel directing anion fluxes to the vacuole [133] so its up-regulation in ripening berries matches the activation of H^+^ pumps counteracting excessive acid decompartmentalisation during ripening [134]. In this study, *VvALMT9* (*VIT_17s0000g03850*, cluster 8) was repressed by short stress during the day in VC, which confirms previous findings in long stress studies [38]. This indicates that the energy-wasting process of malic acid re-entry into the vacuole is transcriptionally repressed by stress, promoting the net release of malic acid at higher temperatures.

Recent research into malic acid focused on dicarboxylate/tricarboxylate transporters (DTCs) belonging to the mitochondrial family (MCF). MCF’s transport different metabolites (di- and tricarboxylates, amino acids, keto acids in addition to nucleotides and coenzymes/cofactors) across the inner mitochondrial membrane [135,136]. DTC’s can transport all the di- and tricarboxylates of the TCA cycle with the exception of fumarate, and they exhibit a high specificity for malate. The expression of two DTCs genes (*VvDTC2* and *VvDTC3*) correlated well with the malic acid content in grape berry mesocarp close to the onset of ripening, and might be involved in the transport of malate into mitochondria [137]. In response to heat treatment, it was found that a large number of *DTC* isogenes, (*VIT_00s0607g00010, VIT_00s0827g00020, VIT_00s0827g00030, VIT_07s0031g02470, VIT_08s0007g07270*; cluster 4; annotated mitochondrial 2-oxaglutarate/malate carrier protein in RefSeq [54]) were induced especially at VS were malic acid respiration occurs at maximal rate, thus implying their putative role in malic acid metabolism. The whole set of transcriptomic data on soluble enzymes and mitochondrial transporters clearly confirms accelerated malate respiration by heat stress in ripening berries.

### Temperature impacts cell wall metabolism differently according to developmental stages

The MapMan graph (Figure 5) indicates a heat induction of transcripts involved in cell wall metabolism, which is less night-specific in G than in VS and VC. Curiously, a large number of transcripts within this category showed heat repression only at VS, where they seem to respond more during the daytime (Figure 5). A more detailed analysis of functional categories enriched by stress (Additional file 3) showed that these cell wall modifications are mainly due to modification of xyloglucan, notably transcripts coding for xyloglucan endotransglucosylases (XETs).

XETs are involved in many processes related to cell wall modification and remodeling. Xyloglucan (XG) is a primary cell wall hemicellulose that coats and cross-links cellulose microfibrils. It is assumed that either breakage of the cross-links or their disconnection from the microfibrils is required to allow the microfibrils to move apart, allowing the wall to expand [138]. XETs can cut and rejoin XG chains, and are therefore considered as a key agent regulating cell wall expansion and loosening. They are believed to be the enzymes responsible for the incorporation of newly synthesized XG into the wall matrix [139,140] which can enable, for instance, fiber elongation in cotton [141].

The large number of temperature-induced *XET* transcripts in green berries can supposedly be explained by the adaptation of berry volume to temperature and the need to render cell walls more flexible. *XET* transcripts did show significant up-regulation in green berries during the night in a previous study [53] which was partly associated with the pronounced diurnal day - night swelling pattern of green berries [142]. Surprisingly, many *XET’*s inverse their heat response at the VS stage and are again heat-induced during VC at night (*VIT_11s0052g01180*; cluster 6, *VIT_05s0062g00610*; cluster 5). Greer and Weston, 2010 [28] observed an inhibition of berry expansion under heat stress which could explain the repression of *XET*s at VS. It has been reported that the resumption of berry growth after véraison slightly lags behind the onset of sugar accumulation [143]; and it is probable that heat treatment slowed down sugar accumulation as was shown in previous studies [28] and delayed thereby the resumption of growth. A strong co-down-regulation of expansin and expansin-like (cell wall modification or remodeling enzymes [144]) transcripts at the onset of ripening supports this hypothesis. Presumably the inversion of *XET* expression by stress throughout development can be attributed to berry elasticity which increases considerably at véraison, where it coincides with a significant drop in turgor [145]. *XET*’s seem be very responsive to the circadian rhythm, temperature and development stage; further time-course and abiotic stress studies are required for a greater understanding of their role in berry development and stress response.

## Conclusions

This study investigated the transcriptomic response of grapevine fruit at three different developmental stages exposed to heat stress during the day or at night. To reduce errors due to berry heterogeneity, sugars and acids were analyzed in each individual berry in order to precisely identify their development stage. In addition, the new microvine model enabled the execution of whole plant experiments in climate chambers controlling experimental conditions to a degree, which was impossible in previous studies.

New clues as to the impact of heat stress on many critical metabolic pathways involved in grapevine fruit development are provided. With precise sampling, deciphering the fruit response both during the day and at night, the obtained findings corroborate field experiments, previous data and empirical observations. New molecular evidence is provided for empirically observed reductions in acidity, aromatic potential and secondary metabolites as a result of elevated temperatures.

The need for strict selection of ripening berries was emphasized by transcripts involved in primary and secondary metabolism pathways (such as malic acid degradation and anthocyanin biosynthesis) for which the heat response was detectable only at the reconstituted véraison stage (VS). The importance of incorporating several time points in such studies was demonstrated by night specific modulation of key enzymes such as *CHS* and *PAL*.

Furthermore, molecular data obtained in this study corroborates the previously reported delay in the onset of ripening due to heat stress. Strikingly, this was only observed immediately after the lag phase, during the reconstituted VS stage, whereas at more advanced stages sugar accumulation seems instead to be favored by high temperatures. Most of the heat stress related transcripts were modulated independently of stage and time whereas some such as the heat stress transcription factor B2B were induced only at night, indicating that no general regulation pattern throughout berry development exists even when same treatments are applied. The magnitude of heat stress-induced transcriptional changes validates the approach used in this study to apply short but intense heat stress to berries, which can often occur under field conditions during summer. The present study provides clues to the transcriptomic adaptation of the berry to heat stress but as expected no major physiological or biochemical changes occurred within the short time of stress application. Therefore long-term studies are required and are underway to validate results from a more physiological point of view.

## Methods

### Plant material

One year-old microvine plants were grown under controlled greenhouse conditions until a whole reproductive cycle from flowering until maturity was obtained along the main axis. Two replicates of six plants were then adapted for one week in two different climate chambers at a constant day - night temperature couple of 22/12°C (Photoperiod: 14 h VPD: 1 kPa). Heat stress was applied 2 hours after sunrise in one cabinet and two hours after sunset in the other. Stress lasted 2 h prior to sampling of one cluster at the green stage and the first three clusters after the lag phase. Seeds were removed from green berries immediately before freezing in liquid N_2_. Berries of clusters after the lag phase were individually wrapped in aluminium foil in order to avoid splitting during freezing. Seeds were removed during N_2_ crushing. Subsequently aliquots of all sampled individual berries were analyzed for organic acids and sugar in order to constitute homogenous batches for RNA extraction (10 berries per triplicate). Sampling and the stress application protocol is illustrated in Figure 1. Control plants were adapted under same conditions and sampled at the corresponding times during the day and at night.

### Organic acid and sugar analysis

Organic acid, glucose and fructose analysis was carried out on approximately 0.1 g of sample powder ground in liquid nitrogen. Samples were diluted five-fold in deionized water and frozen at -20°C. After defrosting, aliquots were heated (60°C for 30 min) homogenized and diluted with 4.375 μM acetate as an internal standard. Sigma Amberlite® IR-120 Plus (sodium form, 0.18 g) was added to 1 mL of sample to prevent potassium bitartrate precipitation. Tubes were agitated in a rotary shaker for at least 10 hours before centrifugation (13000 rpm for 10 min). Supernatants were transferred into HPLC vials before injection on Aminex HPX®87H column eluted under isocratic conditions (0.05 mL.min^-1^, 60°C, H_2_SO_4_) [146]. Organic acids were detected at 210 nm with a waters 2487 dual absorbance detector®. A refractive index detector Kontron 475® was used to determine fructose and glucose concentrations. Concentrations were calculated according to Eyegghe-Bickong *et al*. 2012 [147].

### RNA extraction

RNA extraction was performed as described by Rienth *et al*., 2014 [148]. Briefly: the extraction buffer contained 6 M guanidine-hydrochloride, 0.15 M tri-sodium-citrate, 20 mM EDTA and 1.5% CTAB. Five volumes of room temperature extraction buffer supplemented with 1% MSH were added to 1 g of powder followed by immediate agitation. Cell debris was removed by centrifugation, after chloroform washing, one volume of isopropanol was added to precipitate RNA. Samples are kept at – 20°C for at least two hours. Precipitated RNA was separated by centrifugation after cleaning with 75% ethanol, and the pellet was resuspended in RLC Buffer from the Quiagen rnaEasy® Kit previously supplemented with 1.5% CTAB. To reduce pectin and tannin residues, a second chloroform wash was carried out. The succeeding washing steps and the DNAse treatment were performed as described in the kit. Absorbances at 260 and 280 nm were measured with a NanoDrop 2000c Spectrophotometer Thermo Scientific®. The integrity of RNA was determined using a 2100 Bioanalyzer (Agilent Technolgies®).

### Microarray analysis

cDNA synthesis, labelling, hybridization and washing reactions were performed according to the NimbleGen Arrays User's Guide (V 3.2). Hybridization was performed on a NimbleGen® microarray 090818 Vitis exp HX12 (Roche, NimbleGen® Inc., Madison, WI), containing 29,549 predicted genes representing 98.6% of the 12X grapevine gene prediction version V1 http://srs.ebi.ac.uk/. The chip probe design is available at the following URL: http://ddlab.sci.univr.it/FunctionalGenomics/.

The Robust Multi-array Analysis (RMA) algorithm was used for background correction, normalization and expression levels, [149]. Differential expression analysis was performed with the Bayes t-statistics from the linear models for microarray data (limma) [150]. P-values were corrected for multiple-testing using the Benjamini-Hochberg’s method [151].

Differential expression of genes was analysed between heat stress and control conditions at all developmental stages and time points. Transcripts were considered as significantly modulated when the absolute fold change was > 2 (log_2_ fold change > 1) and the adjusted p value was < 0.05 between heat stress and control at at least one stage and time point. Hierarchical clustering was carried out using the Multiple Experiment Viewer® version 4.6.2, using Pearson’s correlation distance calculated on RMA log_2_ transformed and mean centered gene expression profiles. The raw data is available at the Gene Expression Omnibus (http://www.ncbi.nlm.nih.gov/geo/info/linking.html) under the series GSE53409.

Gene annotation was derived from Grimplet *et al*., 2012 [54]. In order to compare functional annotation, protein sequences of significantly modulated genes were BLASTED against the NCBI RefSeq database [54]. Alignment of sequences was considered as acceptable when the ratio between score and aligned sequence length was superior to 1.6.

Log_2_ changes of day + night and night stage-specific differentially expressed transcripts were integrated using MapMan® software [142,75]. Functional categories were derived from Grimplet *et al*., 2012 [54]. In order to identify significant enrichment of functional categories Fisher’s exact test was carried out to compare the genes list with non-redundant transcripts from the grapevine genome with the FatiGO analysis tool [152]. Significant enrichment was considered in case of p value < 0.01 and illustrated as fold change. To identify the *MBF1c Arabidopsis thaliana* regulon sequences, gene numbers were derived from Suzuki *et al*., 2011 [78] and correspondence was found in the uniprot database (http://www.uniprot.org) and BLASTED against NCBI RefSeq vitis proteins [54]. The first hit was retained and sequences were formatted as per the database. The correspondence with vitis-unique ID gene numbers was obtained by blastx.

### Gene expression validation

cDNA synthesis was performed with ImProm-II TM Reverse Transcription System from Promega®. Quantitative real-time PCR expression analysis was carried out using the StepOnePlus Real Time PCR system (Applied Biosystems®). Twenty μL reaction mixes were prepared, which included 10 μL of iQ™ SYBR Green Supermix (Bio-Rad), 0.5 μM of each primer and 5 μL of diluted cDNA. Gene transcripts were quantified with normalization to VvEF1α as internal standard. All biological samples were tested in triplicate and dissociation kinetics were conducted at the end of each PCR run. The efficiency of each primer pair was measured on a PCR product serial dilution. Quantitative real time q-PCR primers were derived from Cutanda-Perez *et al*., 2009 [97]: VvMybA1 (F: TAGTCACCACTTCAAAAAGG / R: GAATGTGTTTGGGGTTTATC), UFGT (F: GGGATGGTAATGGCTGTGG / R: ACATGGGTGGAGAGTGAGTT), GST (ACTTGGTGAAGGAAGCTGGA / R: TTGGAAAGGTGCATACATGG), *VvanthoMate3* (R: GCAAACAACAGAGAGGATGC / F: AGACCTCGACAATGATCTTAC).

### Anthocyanin analysis

One hundred mg of berry powder that was used for RNA extraction and microarray analysis was used for anthocyanin analysis. Analysis was performed as described in Agorges *et al*., 2006 [153].

## Competing interests

The authors declare that they have no competing interests.

## Authors’ contributions

MR and CR conceived and designed the experiments, analyzed and interpreted the data and wrote the manuscript. NL and RC participated at plant culture. LT, MK and DL participated in discussion of results and paper corrections. All authors read and approved the final manuscript.

## Supplementary Material

Additional file 1 PC2 vs PC 4 of principal component analysis on normalized expression data. Click here for file

Additional file 2 Heat stress-modulated genes. Click here for file

Additional file 3 Functional categories of heat stress induced transcripts separately analyzed in all developmental stages at day and night. Click here for file

Additional file 4 Functional categories of heat stress repressed transcripts separately analyzed in all developmental stages at day and night. Click here for file

Additional file 5 **Enriched functional categories over-represented in each cluster (1-8).** Values are illustrated as fold change of each significantly (p < 0.05) enriched category when compared to non-redundant transcripts from the grapevine genome. Click here for file

Additional file 6 Real-time q-PCR validations of anthocyanin biosynthesis-related transcripts. Click here for file
