# Supplementary figures and images for: Day and night heat stress trigger different transcriptomic responses in green and ripening grapevine (vitis vinifera) fruit
Source: BMC Plant Biol. 2014 Apr 28;14:108. doi: 10.1186/1471-2229-14-108 (PMC4030582; doi:10.1186/1471-2229-14-108)

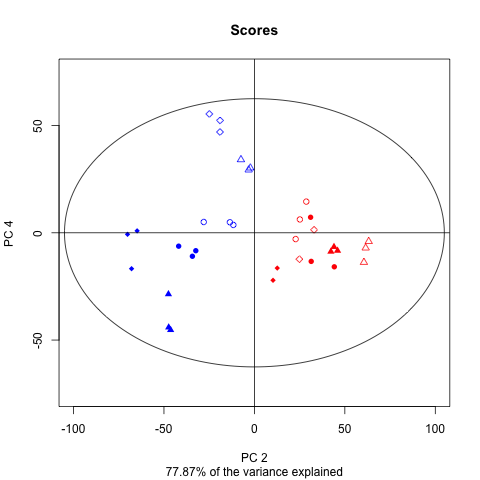

Supplement: Additional file 1 — PC2 vs PC 4 of principal component analysis on normalized expression data. [file 1471-2229-14-108-S1.tiff]

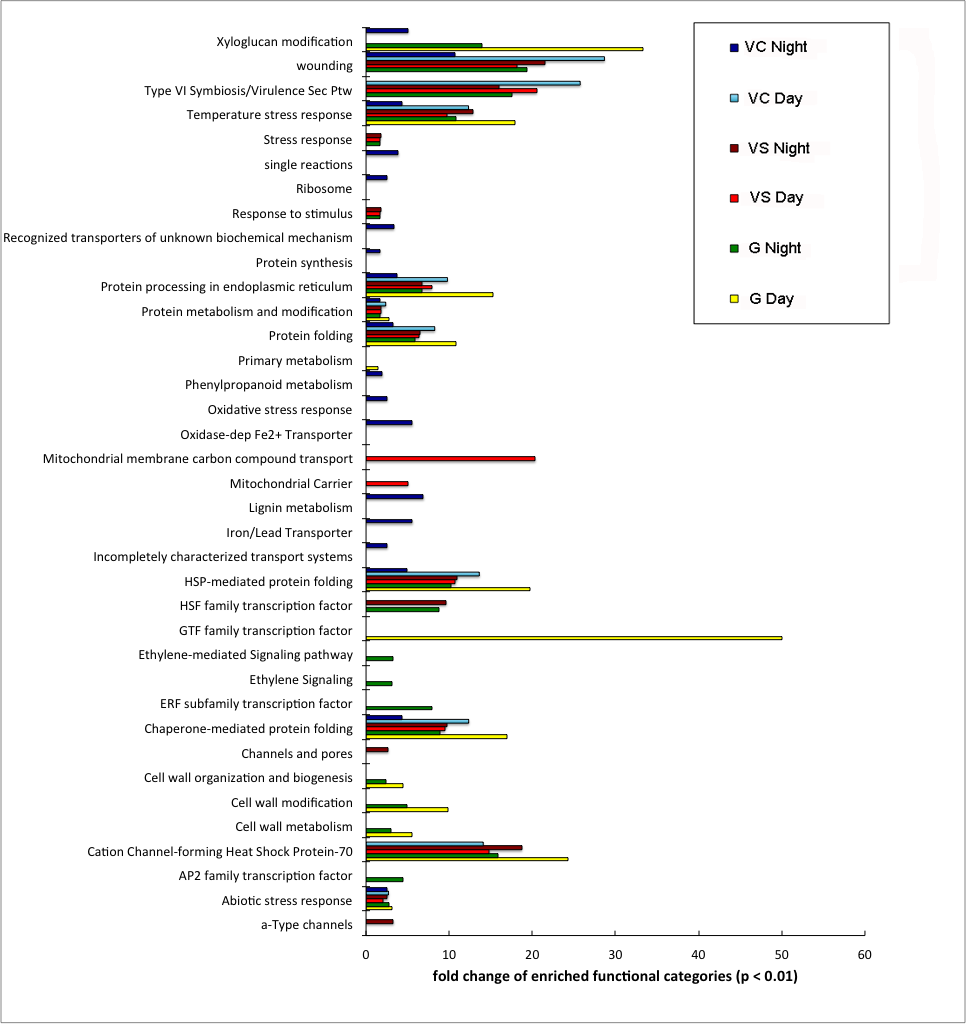

Supplement: Additional file 3 — Functional categories of heat stress induced transcripts separately analyzed in all developmental stages at day and night. [file 1471-2229-14-108-S3.tiff]

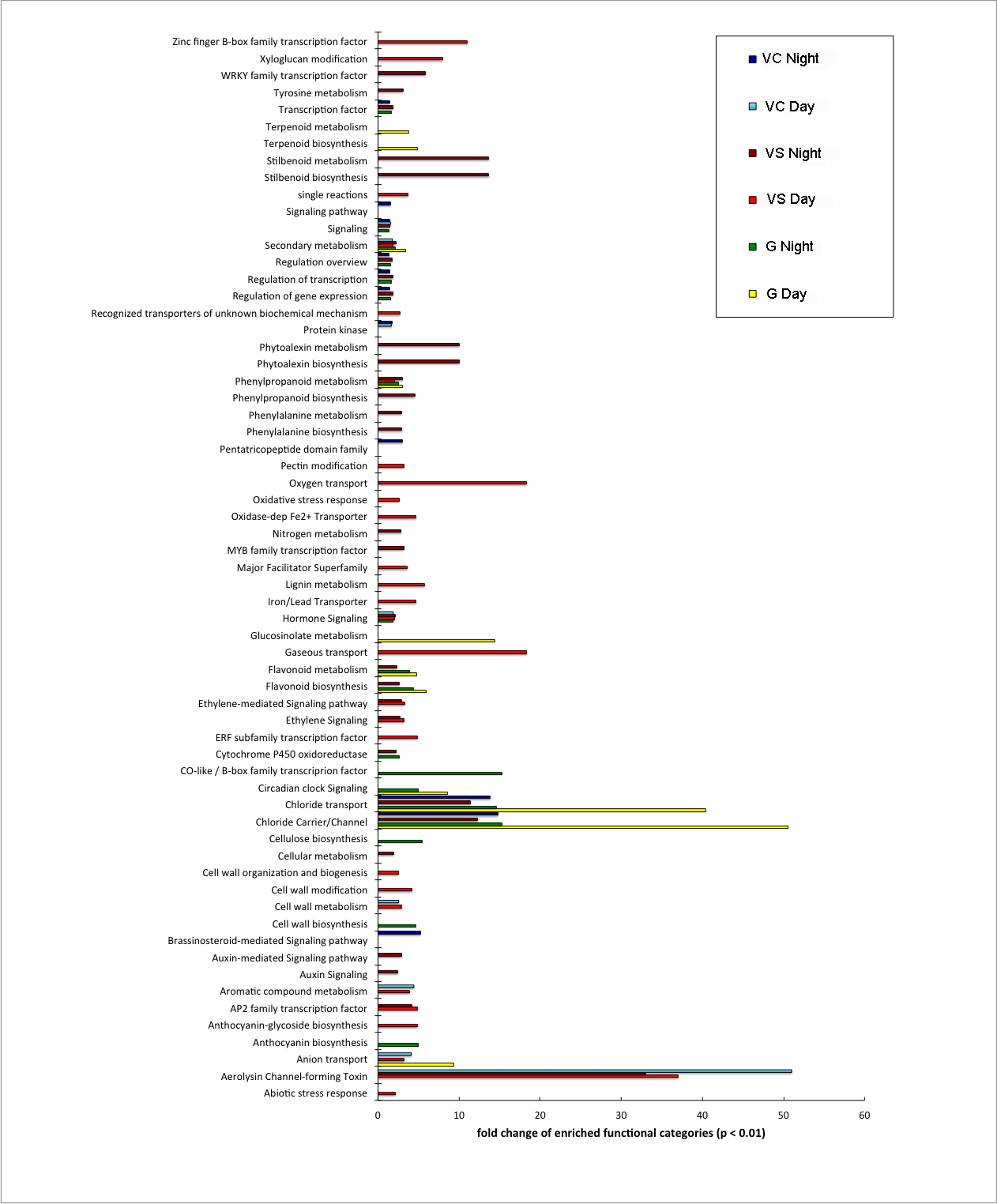

Supplement: Additional file 4 — Functional categories of heat stress repressed transcripts separately analyzed in all developmental stages at day and night. [file 1471-2229-14-108-S4.tiff]

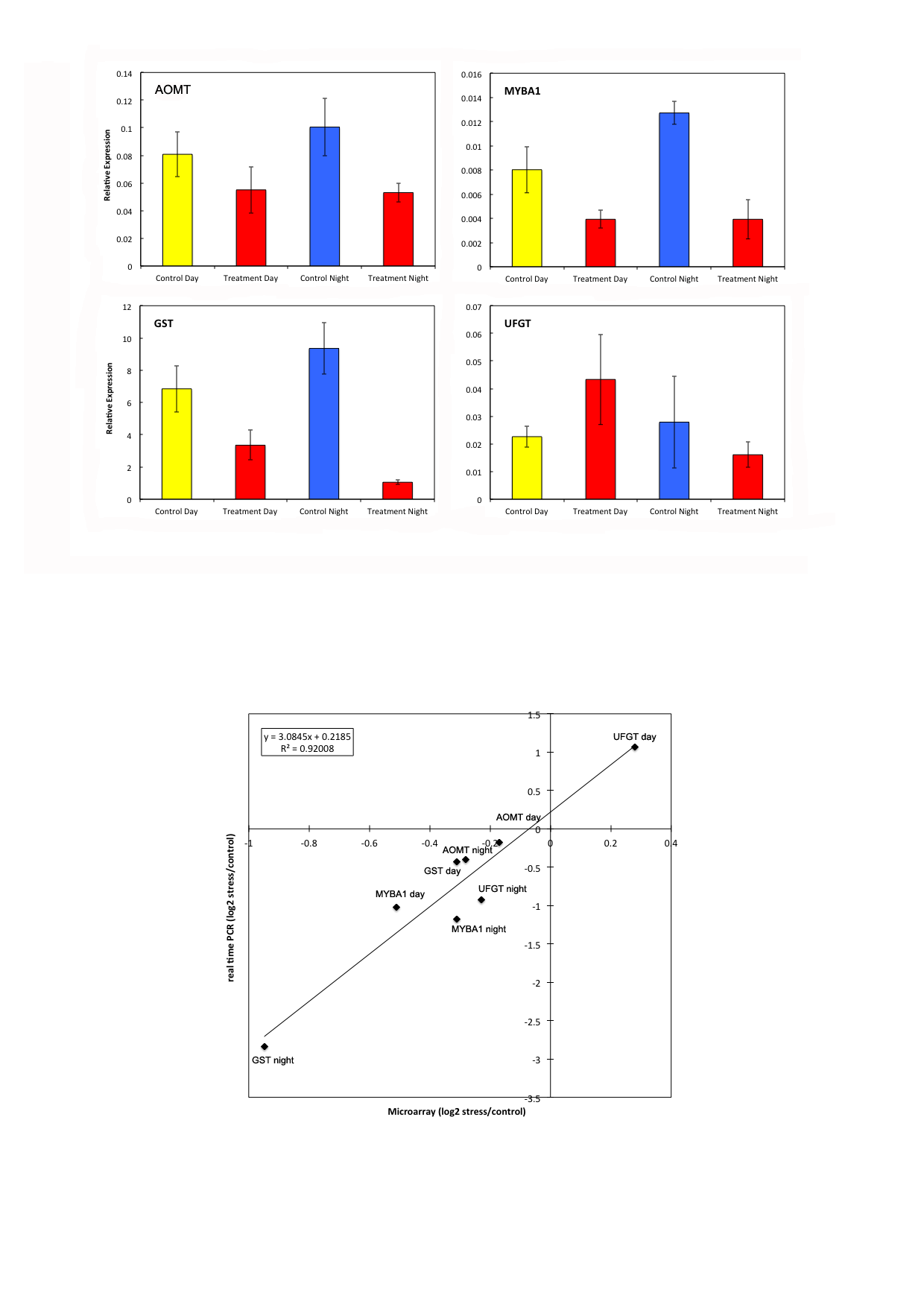

Supplement: Additional file 6 — Real-time q-PCR validations of anthocyanin biosynthesis-related transcripts. [file 1471-2229-14-108-S6.tiff]
