# Supplementary material for: Day and night heat stress trigger different transcriptomic responses in green and ripening grapevine (vitis vinifera) fruit
Source: BMC Plant Biol. 2014 Apr 28;14:108. doi: 10.1186/1471-2229-14-108 (PMC4030582; doi:10.1186/1471-2229-14-108)
Supplement: Additional file 5 — Enriched functional categories over-represented in each cluster (1-8). Values are illustrated as fold change of each significantly (p < 0.05) enriched category when compared to non-redundant transcripts from the grapevine genome. [file 1471-2229-14-108-S5.pdf]

CLUSTER					1	2	3	4	5	6	7	8
**Transport Overview**	Gaseous transport				46							
		oxygen transport			46							
	channels and pores	a-type channels	annexin			20						
			Cat. channel-form, HSP-70					12				
	Incompl. charact. transp.syst.											3
		unkwon bioch. Reactions										3
			Iron / lead transp.									5
				Ocidase-dep Fe2+ tr								5
	Mitoch. Mem. transp.							15				
**Signaling**											1.5	
	hormone signaling										1.5	
		ethylene signaling									2.4	
			ethylene-med. sign.								2.5	
	signaling pathway	protein kinase									1.6	
**Response to stimulus**	Stress response	Abiotic stress										2.2
			Oxidative stress response									2.5
			Temp. stress response					7.1				
			wounding									9.8
**Regulation of gene expression**											1.4	
	Regulation of transcription										1.4	
		Transcription factor									1.4	
			Co-like/B-box family					16.4				
			AP2 family						5.7			
				ERF subfamily					1.5			

CLUSTER					1	2	3	4	5	6	7	8
**Primary metabolism**	Amino acid metabolism	Aromatic amino acids	Tyrosine metabolism									
			Phenylalanine metabolism									
				Phenylalanin biosynthesis								
	Protein metabolism and modification	Protein processing in endopl. retic.				7.8						
		Protein folding				5.2		3.0				1.9
			Chaperone-med. prot. fold.			7.6		4.6				2.6
				HSP-med. Prot. folding		8.4		4.7				2.6
		Protein synthesis	Ribosome								2	
	Carbohydrate metabolism	Oligosacch. metab	Trehalose metabolism								3.2	
**Secondary metabolism**							2.3					
	Terpenoid metabolism						2.6					
**Phenylpropanoid metabolism**							2.6					
	Lignin metabolism											6.7
	Phenylprop. biosythn.										5.0	
	Stilbenoid metabol.										15.4	
	Flavonoid metabolism						4.0				2.4	
		Silbenoid biosynthesis									15.4	
		Flavonoid biosynth.					4.5				2.7	
			Flavon.-glycosid biosynth.							36		
			Anthocyanin biosynthesis				6.8					
				Anthocy.glyco.biosynth.			6.2					
**Cellular component organization & biogenesis**										2.7		
	Cell wall org.and biogen								3.2	7.0		
		Cell wall metab							3.7	7.3		
			Cell wall modif.						4.2	12.9		
				Xyloglucan modif.						44.5		
**Metabolism**	Cellular metabolism										2	
		Phytoalexin metabl.									10.9	
			Phytoalexin biosynth.								10.9	
		Nitroh. domain family	Nitrogen metabolism								3.3	
**Diverse function**	Pentatricopeptid domain family										2.5	
